# Snaplage residue as a fiber source for finishing beef cattle under grazing: effects on residue characteristics, cattle behavior and performance, and carcass traits

**DOI:** 10.1093/tas/txae173

**Published:** 2024-12-09

**Authors:** Italo B G de Lima, Priscila J R Cruz, Lucas P C Borges, Mateus P Gionbelli, Marcio M Ladeira, Daniel W Shike, Daniel R Casagrande, Thiago F Bernardes

**Affiliations:** Department of Animal Science, University of Lavras, Lavras, 37200-900, Brazil; Department of Animal Science, University of Lavras, Lavras, 37200-900, Brazil; Department of Animal Science, University of Lavras, Lavras, 37200-900, Brazil; Department of Animal Science, University of Lavras, Lavras, 37200-900, Brazil; Department of Animal Science, University of Lavras, Lavras, 37200-900, Brazil; Animal Sciences Laboratory, University of Illinois at Urbana-Champaign, Urbana, IL, 61801, USA; Department of Animal Science, University of Lavras, Lavras, 37200-900, Brazil; Department of Animal Science, University of Lavras, Lavras, 37200-900, Brazil

**Keywords:** beef cattle, crop-livestock integration, grazed corn residue, snaplage residue

## Abstract

The snaplage residue presents itself as a potential alternative roughage source in finishing systems, owing to its high fiber concentration which aids in maintaining rumen health. Nevertheless, the performance of animals will hinge on both the allowance and the nutritive value it offers. This study aimed to evaluate different stocking rates of heifers grazing snaplage residue as an exclusive source of fiber on finishing phase performance. The treatments included two stocking rates (SR): i) low stocking rate (LS; 3.5 AU/ha) and ii) high stocking rate (HS; 7.0 AU/ha), which were obtained by modifying the size of the paddocks. Crossbred beef heifers (*n* = 48; initial body weight = 276 ± 23 kg) were assigned to 16 paddocks (3 heifers/paddock). The concentrate (87% of corn, 3.5% of soybean meal, 3.9% of cottonseed meal, 1.2% of urea, and 4% of mineral; DM basis) was fed ad libitum daily at 0600 hours. Data were analyzed using the MIXED procedure of SAS. There was no SR × time effect (*P* = 0.88) on residue mass. There was less (*P* < 0.01) total residue mass for HS than LS, and total mass decreased (*P* < 0.01) over time. There was no SR × time effect (*P* ≥ 0.16) for behavior characteristics. There was no difference between HS and LS for average residue intake (*P* = 0.34; 0.44 vs 0.48 kg/d, respectively), concentrate intake (*P* = 0.84; 7.72 vs 7.78 kg/d, respectively), and daily gain (ADG; *P* = 0.94; 0.95 vs 0.95 kg/d, respectively), The HS treatment increased (*P* < 0.01) gain per area (618 vs 309 kg/ha) compared to LS. No differences between SR were observed for carcass characteristics (*P* ≥ 0.12*).* The meat’s chemical composition was not different (*P* ≥ 0.37) between treatments. Overall, the snaplage residue stocking rate did not affect the finishing phase performance of beef heifers, but the greater stocking rate (7.0 AU/ha) increased gain per land area.

## Teaser

This study highlights snaplage residue as a promising fiber source for finishing beef cattle, demonstrating that while different stocking rates did not significantly affect animal performance, a higher stocking rate improved weight gain per area. This finding suggests that farmers can optimize land use without compromising cattle growth or meat quality.

## Introduction

Snaplage, which contains the ear (cob and kernels), husk, and shank (Ferraretto et al., 2018), is increasingly being adopted in Brazilian feedlots (Gusmão et al., 2021; Bernardes et al., 2024). Currently, snaplage accounts for one-third of beef cattle producers who process grains (Bernardes et al., 2022). When snaplage is harvested, a substantial residue, composed of leaves and stem of the corn plant, remains in the field. In Brazil, this residue has been generally used as a ground cover for the subsequent no-till cropping practice. In the United States, grazing corn crop residue by cattle is a management practice used mainly in the Corn Belt and the Midwest (Sulc and Tracy, 2007; Redfearn et al., 2019; Lehman et al., 2021). Grazing corn crop residue is a low-cost feeding strategy for backgrounding and cow-calf operations, especially in the fall and winter (Lehman et al., 2021).

Livestock production in feedlots has been widely adopted worldwide. However, operational and structural costs have led several beef cattle producers to adopt the grass-finishing system (GFS; Mota et al., 2020). Such a system is a common management practice among Brazilian farms (Mota et al., 2020), especially in regions where there is a high availability of co-products and concentrate feeds. This practice is based on providing levels of concentrate like those adopted in feedlots to grazing animals. Thus, concentrate becomes the main source of nutrients in the diet. In this system (GFS), animals receive between 1.5% and 2% of BW in concentrate (Mota et al., 2020). At this level of supplementation, a substitution effect occurs, in which the animal consumes less forage, allowing for increased stocking rates (Moore et al., 1999; Paterson et al., 1994). In traditional GFS, stocking rates are adjusted based on the estimated productivity of the available forage (Rouquette, 2015; Congio et al., 2019), and it is possible to modulate forage growth dynamics using nitrogen fertilization (Homem et al., 2021). This strategy allows for a constant quantity and quality of forage canopy without affecting animal performance. It could also allow adding animals and increasing the gain per land area. However, grazing corn crop residue has a different dynamic than grazing a growing forage due to losses in quantity and quality of residue by intake, trampling, and weathering (Russell et al., 1993; Stalker et al., 2015; Lehman et al., 2021)

Considering that stocking rates in the conventional GFS can vary during the seasons, from 5 to 6 AU/ha in the dry season and up to 10 AU/ha in the rainy season, a stocking rate of 7.0 AU/ha was considered an average stocking rate adopted in conventional GFS (Mota et al., 2020). However, due to scarce literature on grazing snaplage residue in tropical environments, half the stocking rate (3.5 AU/ha) was adopted for snaplage residue finishing system (SRFS) to understand the effects of these stocking rates on the dynamics of residue disappearance in quality and quantity and animal productivity. Thus, we hypothesized that greater stocking rates on snaplage residue could negatively affect animal performance and meat quality due to a reduction in the availability of residue in the diet of finishing animals resulting in a higher risk of acidosis. However, increasing the stocking rate may promote greater gain per land area. Therefore, our objective was to investigate the effects of snaplage residue stocking rate on characteristics of snaplage residue, ingestive behavior, animal performance, and carcass traits.

## Materials and Methods

The experimental procedures of this study were approved by the Ethics and Animal Welfare Committee of the Federal University of Lavras (protocol number 008/2019).

### Experimental Site and Treatments Establishment

**Figure 1.**
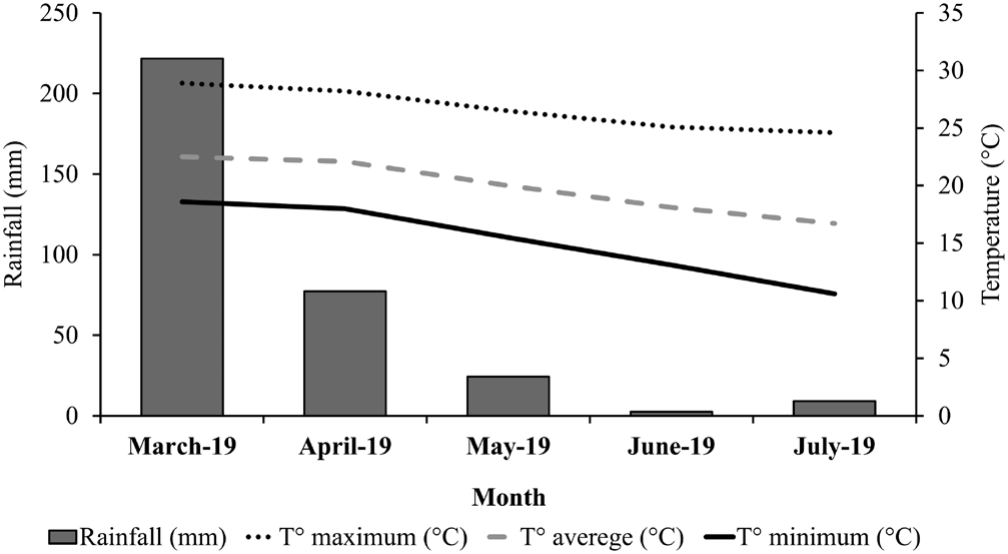
Mean monthly temperatures and rainfall in Lavras, MG, Brazil, during the experimental period.

The experiment was conducted at the Experimental Farm of the Federal University of Lavras, Brazil (21°14ʹ09″ S, 45°58ʹ35″ W; 880 m above sea level). This area has a subtropical humid mesothermal climate with dry winters (Köppen-Geiger climate classification: Cwa; Sá Júnior et al., 2012). Meteorological data (Figure 1) were obtained from a weather station located 1,000 m from the experimental area.

**Figure 2.**
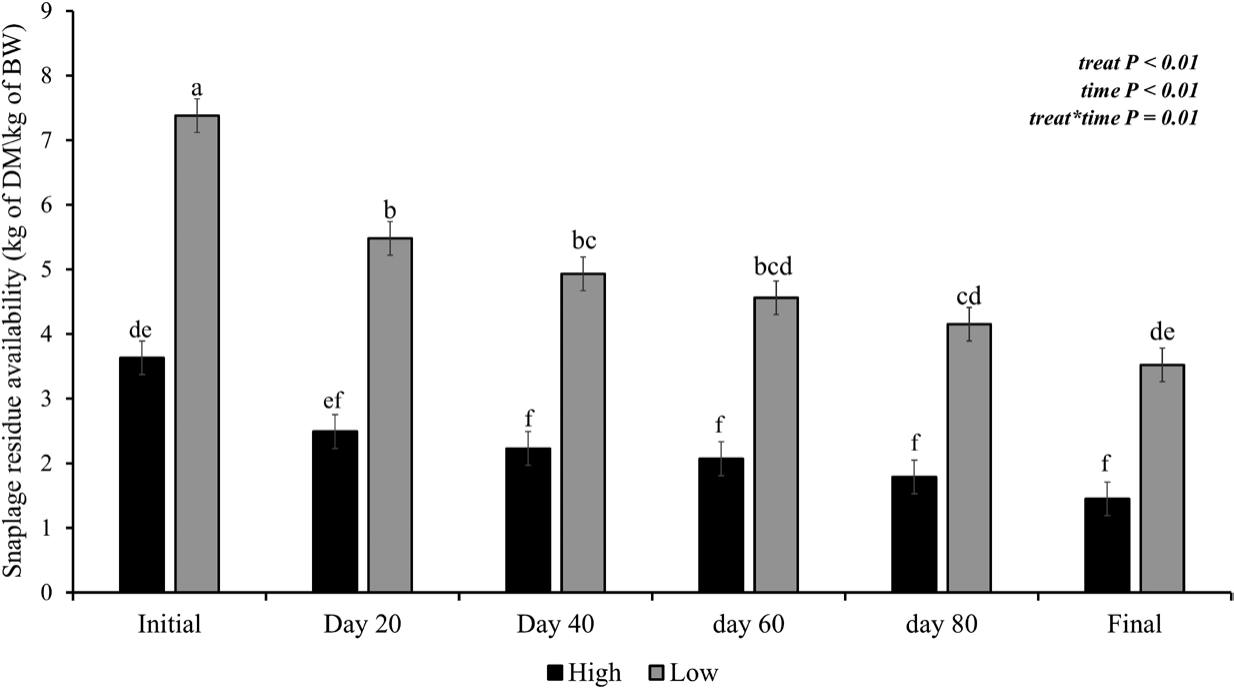
Effects of stocking rate and period (0, 20, 40, 60, 80, and 96 d) on snaplage residue availability; LS, low stocking rate (3.5 AU/ha); HS, high stocking rate (7.0 AU/ha). Error bars represent the SEM.

Immediately after the snaplage harvest, by using a self-propelled forage chopper equipped with a snapper head, the area was divided into 16 paddocks, and the treatments were randomly allocated. The treatments were stocking rates (SR) in the animal unit (AU; the animal unit was considered a bovine weighing 500 kg; Allen et al., 2011), namely: i) low stocking rate (LS; 3.5 AU/ha) and ii) high stocking rate (HS; 7.0 AU/ha). The treatments were obtained by modifying the size of the paddocks for each treatment. The LS and HS paddock sizes were 0.6 and 0.3 ha, respectively, with an initial forage availability of 7.4 and 3.7 kg of DM/kg of BW, respectively (Figure 2). Forty-eight single-sourced beef heifers (Angus × Nellore; 276 ± 23 kg of body weight [BW] and 16 ± 1.3 mo of age) were procured from a local Lavras auction facility and received 3 wk prior to study initiation. During those 3 wk, heifers were offered a common *Brachiaria* pasture. On the day of experiment initiation, heifers were identified, treated against internal and external parasites (Solution® 3,5% - MSD Animal Health), weighed, and entered the experimental area (3 heifers/paddock). The grass-finishing phase lasted 96 d. The chemical composition of the snaplage residue was determined on day 20 of the experimental period and is described in Table 1. The concentrate was fed ad libitum daily in the morning at 0600 hours and its chemical composition is displayed in Table 2.

**Table 1.** Effect of stocking rate of finishing heifers grazing snaplage residue and fed ad libitum concentrate on nutrient composition of the residue components at day 20 of the experimental period

Item	Treatments^†^		SEM^*^	*P-value*
	HS	LS		
Leaf				
Organic matter, % DM	85.7	87.4	1.44	0.26
apNDF^‡^, % DM	63.4	66.1	1.81	0.17
iNDF^‖^, % DM	38.2	36.3	2.66	0.50
Crude protein, % DM	5.84	6.07	0.35	0.54
Sheath				
Organic matter, % DM	92.9	94.1	0.73	0.13
apNDF^‡^, % DM	75.0	77.3	2.69	0.40
iNDF^‖^, % DM	36.3	39.7	2.93	0.27
Crude protein, % DM	3.96	4.38	0.31	0.20
Stem				
Organic matter, % DM	95.5	96.0	0.4	0.26
apNDF^‡^, % DM	82.2	82.4	1.7	0.94
iNDF^‖^, % DM	46.6	44.6	1.63	0.25
Crude protein, % DM	2.89	2.89	0.11	0.98

^*^Standard error of the mean.

^†^Treatments: HS (High stocking rate; 7.0 AU/ha), LS (Low stocking rate; 3.5 AU/ha).

^‡^apNDF: Neutral detergent fiber corrected for ash and protein.

^‖^iNDF: Indigestible Neutral detergent fiber (incubated in the rumen for 288 h).

NDF, neutral detergent fiber.

**Table 2.** Ingredient and nutrient composition on a dry matter basis of the concentrate fed ad libitum to heifers grazing snaplage residue

Item	Concentrate^*^
Ingredients, %	
Corn	87.0
Soybean meal	3.5
Cottonseed meal	3.9
Urea	1.6
Mineral	4.0
Nutrient concentration	
Dry matter, %	90.0
Organic matter, % DM	90.6
Neutral detergent fiber, % DM	3.4
Indigestible neutral detergent fiber^†^, % DM	3.9
Crude protein, % DM	16.6
Starch, % DM	75.7
Ether extract, % DM	3.9

^*^Concentrate contained: 74.7% NDT, 0.86% Ca, 0.32% P, 0.36% Na, 0.49% K, 0.39% Mg, 0.68% S, 15.00 mg/kg Cu, 43.00 mg/kg Mn, 62.00 mg/kg Z, 1.11 mg/kg Co, 0.70 mg/kg I, 0.25 mg/kg Se, 16.78 mg/kg Fl, 3.470.00 IU/kg vitamin A, 360.00 IU/kg vitamin D3, 54.000 IU/kg vitamin E, 23.40 mg/kg monensin sodium.

^†^Indigestible Neutral detergent fiber (incubated in the rumen for 288 h).

### Snaplage Residue Mass

After snaplage harvest, the field was sampled for characterization and quantification of the initial snaplage residue mass in each paddock and its sampling was repeated every 20 d. Three samples were collected in each paddock at randomly selected locations, using frames measuring 1.5 × 1.4 m. After the collection of the snaplage residue, morphological separations were performed. The snaplage residue samples were separated into leaf, stem, and sheath. The samples were oven-dried at 55 °C for 72 h to a constant weight. The snaplage residue mass was considered the sum of the morphological components of the corn. The forage availability was calculated considering the snaplage residue mass (kg/d of DM) divided by the average daily gain regression estimated by the BW (kg).

### Nutritive Value of Feeds

The nutritive value characterization was performed at the same time as the collection period for snaplage residue intake. A composite sample of each morphological component for each experimental unit was made. The snaplage residue and concentrate composite samples were ground in a Cyclotec mill (Tecator, Herndon, VA) to pass a 1-mm screen. After grinding, the dry matter (DM) of each sample was obtained by oven drying at 100 °C for 18 h (Method 934.01; AOAC, 2000). The crude protein (CP) concentration was calculated based on the N concentration (CP = total *N* × 6.25), which was determined using the Kjeldahl procedure (Method 920.87; AOAC, 2000). In the snaplage residue samples, the ash and protein-free neutral detergent fiber (apNDF) were determined by the autoclave method at 105 °C for 60 min (Pell and Schofield, 1993).

In the concentrate samples, ash and protein-free neutral detergent fiber (apNDF) was determined by filter bottom crucible (Method 978.10; AOAC, 2000), and the ether extract (EE) was analyzed according to (Method 920.39; AOAC, 2000). Starch concentration in the concentrate was measured according to an enzymatic method (Hall and Mertens, 2008), with thermostable α-amylase (Ankom Tchnology, Macedon, NY) and amyloglucosidase (Megazyme E-AMGDF, Bray, Co. Wicklow, Ireland).

### Animal Behavior

Heifer behavior was assessed through visual observation on days 15, 30, 45, 60, 75, and 94. Trained observers collected data every 5 min over a 12-h period each day (Johnson and Combs, 1991). The behavioral variables collected were: grazing time, rumination time, feeding time at the bunk, and time in other activities (idleness).

### Diet Intake, Nutrient Intake, and Total Nutrient Digestibility

The individual intake of residue and concentrate was estimated on day 25 using fecal excretion and indigestible neutral detergent fiber (iNDF). Spot fecal samples were collected, and a composite sample was created for each animal for 3 days of collection. On the sampling days, heifers were moved from the paddocks to a barn to collect feces directly from the rectum at times 0700, 1200, and 1700 hours, respectively. Titanium dioxide (TiO_2_) was used to estimate the fecal excretion of animals. Ten grams of TiO_2_ were wrapped in paper cartridges and provided daily to each animal through an esophageal probe. Chromic oxide (Cr_2_O_3_) was used to estimate the individual concentrate intake. The chromic oxide was included in the concentrate (0.15%) consumed by heifers. The chromic and titanium were provided for nine consecutive days, six for adaptation, and three for collection (Titgemeyer et al., 2001). Fecal samples were oven dried at 55 °C for 72 h to determine the DM concentration and air equilibrated, weighed, and ground in a Cyclotec mill (Tecator) to pass 2 and 1-mm screen (2-mm for iNDF analyses and 1-mm for other analyses). The fecal samples were analyzed for titanium dioxide and chrome oxide concentration, according to Myers et al. (2004) and Kimura and Miller (1957), respectively.

Fecal, snaplage residue mass, and concentrate samples were incubated in the rumen for 288 h to determine iNDF (Huhtanen et al., 1994). Two cannulated heifers fed palisadegrass and corn-based concentrate (80:20) were used for iNDF estimation. Fecal excretion was used to determine the total amount of iNDF in feces. Thus, the estimated intake of iNDF per day was obtained. After that, iNDF from the diet (snaplage residue + concentrate) was sampled to estimate the intake. The snaplage residue intake (SRI, kg/d) was obtained using indigestible neutral detergent fiber (iNDF) as the internal marker, as follows:


$$\begin{array}{lll} SRI\;(kg/d)\;=\; & \;[(FE\times iNDF\;in\;the\;feces)-iNDF\;in\;the\;concentrate]\;\; & /\;iNDF\;in\;the\;snaplage\;residue \end{array}$$ (1)

where SRI is the snaplage residue intake (kg/d), FE is the fecal DM excretion (kg/d), iNDF in the feces is the iNDF concentration in the feces (g/kg of fecal DM), iNDF in the concentrate is the amount of iNDF that came from the concentrate (kg/d), and iNDF in the snaplage residue is the iNDF concentration in the snaplage residue (g/kg DM). The total intake was obtained by the sum of the snaplage residue and concentrated daily intake.

The concentrate intake was obtained using chromium oxide (CrO2), by the equation:


$$\begin{array}{l} CI(kg/d)\;=\;[(FE\times ICF)]\;/\;ICC \end{array}$$ (2)

where CI is the Concentrate intake (kg/d), fecal DM excretion (kg/d), ICF is the indicator concentration in the feces (g/kg DM), ICC is the indicator concentration in the concentrate (g/kg).

The nutrient intake was calculated by snaplage residue and concentrate intake multiplied by their respective nutritive value. The coefficients of apparent total tract digestibility of DM, OM, CP, EE, NDF, and starch were calculated using fecal excretion of the external TiO_2_ marker. The DM, OM, CP, EE, NDF, and starch concentrations of fecal samples were determined in the same way as described for the snaplage residue and concentrate nutritive value analyses. The apparent total tract digestibility (g/kg) was calculated as (% DM and nutrients in the diet − % DM and nutrients in feces)/(%DM and nutrients in diet). The apparent digestibility coefficients were calculated for DM, OM, CP, NDF, starch, and EE.

### Animal Performance and Carcass Traits

Heifers were weighed at the beginning (day 0) and at the end of the experimental grazing period (day 96) to determine the average daily gain (ADG), which were performed with 12 h fasting of feed and water.

After 96 d, heifers were slaughtered in a commercial slaughterhouse using captive bolt stunning and jugular vein bleeding, followed by skinning and evisceration. The carcasses were then divided longitudinally into two halves to obtain the hot carcass weight and hot carcass yield. After 24 h of refrigeration at 2 °C, the subcutaneous fat thickness was measured between the 12th and 13th ribs of the left carcass using a graduated caliper at three-fourths of the length of the ribeye from the cranial portion. The longissimus muscle area was also measured between the 12th and 13th ribs, outlined on transparency paper, and quantified after reading by the LI-3100 area meter (LI-COR Inc., Lincoln, Nebraska, USA).

A 2.54 cm-thick steak of the longissimus muscle was removed from the left carcass cranially from the 13th rib for intramuscular fat analysis. After collecting at the packing plant, samples were transported and stored at −20 °C until analysis. The proximate composition analysis was performed on a 100 g ground steak after removing subcutaneous fat, using Foodscan equipment (FOSS, Hillerod, Denmark) using the near-infrared range according to AOAC (2007).

Two steaks were used for a different maturation time (0, immediately after slaughter and 14 d postmortem) on which the CIE color index analyses were performed. The steaks were removed from the vacuum packaging for exposure to oxygen for 30 min. Meat surface reflectance data were recorded from the average of five consecutive measurements using a CM-700 colorimeter spectrophotometer (Konica Minolta Sensing Inc., Osaka, Japan), with an aperture of 8 mm, illuminant D65, 10° observer angle, and in specular component exclusion mode (SCE). From the readings obtained in SCE mode, brightness (L*), redness (a*), yellowness (b*), hue angle (h), and chroma (C*) values were determined. The cooking loss was performed on a grill until an internal temperature of 71 °C was reached, which was monitored using a handheld digital thermometer. After cooking, the steaks remained at room temperature until the temperature stabilized and were subsequently weighed (AMSA, 1995). Total cooking loss was calculated as the difference between the weight of the steaks before and after oven-broiling.

## Statistical analysis

The experiment was carried out in a completely randomized design with two stocking rates (low and high stocking rates) and eight replications. The carcass characteristics, diet digestibility, meat chemical composition, nutritive value, nutrient intake, and performance variables were analyzed using the MIXED procedure of SAS 9.4 statistical software (SAS Inst. Inc., Cary, NC), considering the paddock as the experimental unit. The stocking rate (treatment) was considered as a fixed effect. The statistical model for data analysis was calculated as follows:


$$\begin{array}{l} Yi\;=\;\mu \;+\;SRi\;+\gamma i \end{array}$$ (3)

where *Yi* is the observed measurement in the *i*th stocking rate; *μ* is the overall mean; *SRi* is the fixed effect associated with *i*th stocking rate, i = 1, 2; *γi* is the random error associated with the *i*th SR.

The animal behavior, snaplage residue mass, chemical composition, and meat quality were analyzed by the MIXED procedure (mixed models) of the SAS 9.4 statistical software using repeated measurements over time (evaluation periods). The effects of stocking rate and evaluation periods were considered fixed effects and paddock (treatment) was considered a random effect. The Akaike information criterion was used to choose the best (co)variance structure (Akaike, 1974). All variance components were estimated using the restricted maximum likelihood method. Treatment means were estimated using the LS MEANS statement and compared using the Tukey test. When the interaction was significant, it was sliced by time. Significance was declared at *P* ≤ 0.05 and trends when 0.05 < *P* ≤ 0.10. The statistical model for data analysis was as follows:


$$\begin{array}{l} Yiz\;=\;\mu \;+\;SRi\;+\gamma i\;+\;EPz\;+\;(SR\times EP)iz\;+\varepsilon iz \end{array}$$ (4)

Where Y*iz* is the observed measurement *i*th stocking rate of the *z*th evaluation periods; μ is the overall mean; SR*i* is the fixed effect associated with *i*th stocking rate, *i* = 1, 2; y*i* is the random error associated with the *i*th SR nested within replication; EP*z* is the fixed effect associated with *z*th evaluation periods; (SR × EP)*iz* is the fixed effect of interaction *i*th SR with the *z*th EP; *εiz* is the random error associated with the *i*th SR, and the *z*th EP.

## Results

### Snaplage Residue Mass and Availability

**Figure 3.**
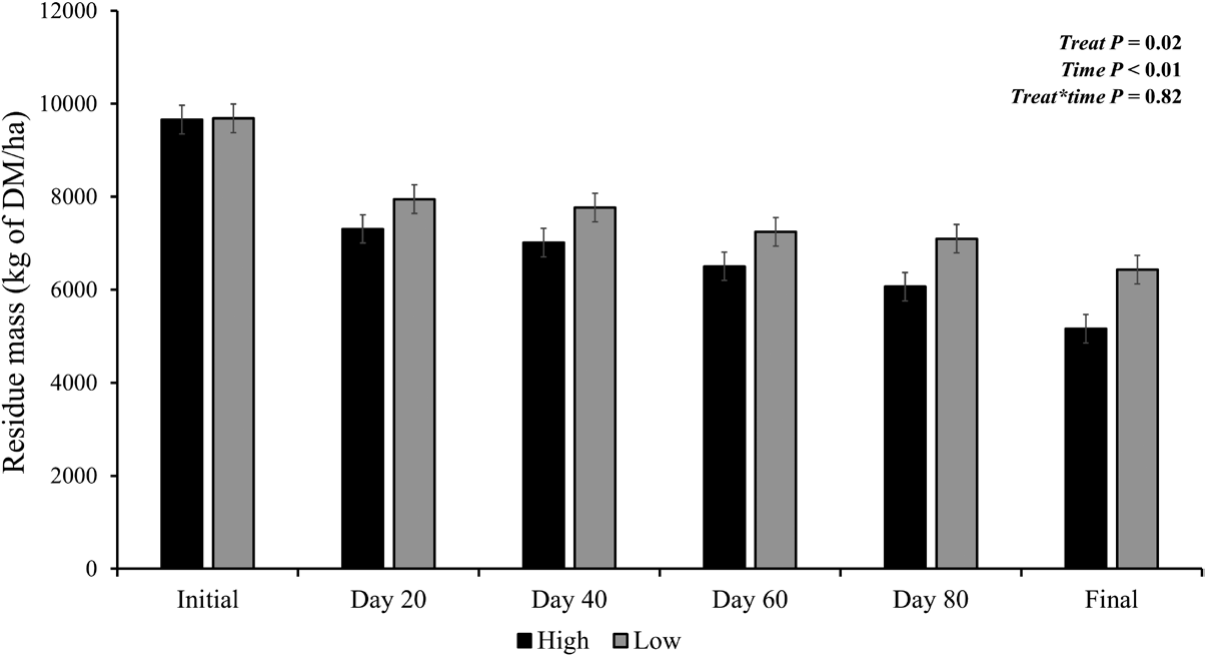
Effects of stocking rate and period (0, 20, 40, 60, 80, and 96 d) on snaplage residue mass; LS, low stocking rate (3.5 AU/ha); HS, high stocking rate (7.0 AU/ha). Error bars represent the SEM.

There was no stocking rate × time effect (*P* = 0.82; Figure 3) on the residue mass. However, an SR effect for total mass was detected (*P* < 0.02) with less total mass for HS than LS (6,951 vs 7,696 kg of DM/ha, respectively). There was also a time effect (*P* < 0.01) with total mass decreasing over time (*P* < 0.01) from 9,659 to 5,162 kg of DM/ha in the HS, and from 9,685 to 6,434 kg of DM/ha in the LS.

There was a stocking rate × time interaction for snaplage residue availability (*P* = 0.01; Figure 3). There was an SR (*P* < 0.01) and time effect (*P* < 0.01). The snaplage residue availability decreased over time in both treatments. The HS had between 41% and 49% less snaplage residue availability than LS. The initial snaplage residue availability was 7.4 and 3.7 kg of DM/kg of BW and the final 3.5 and 1.5 kg of DM/kg of BW (LS and HS, respectively).

**Figure 4.**
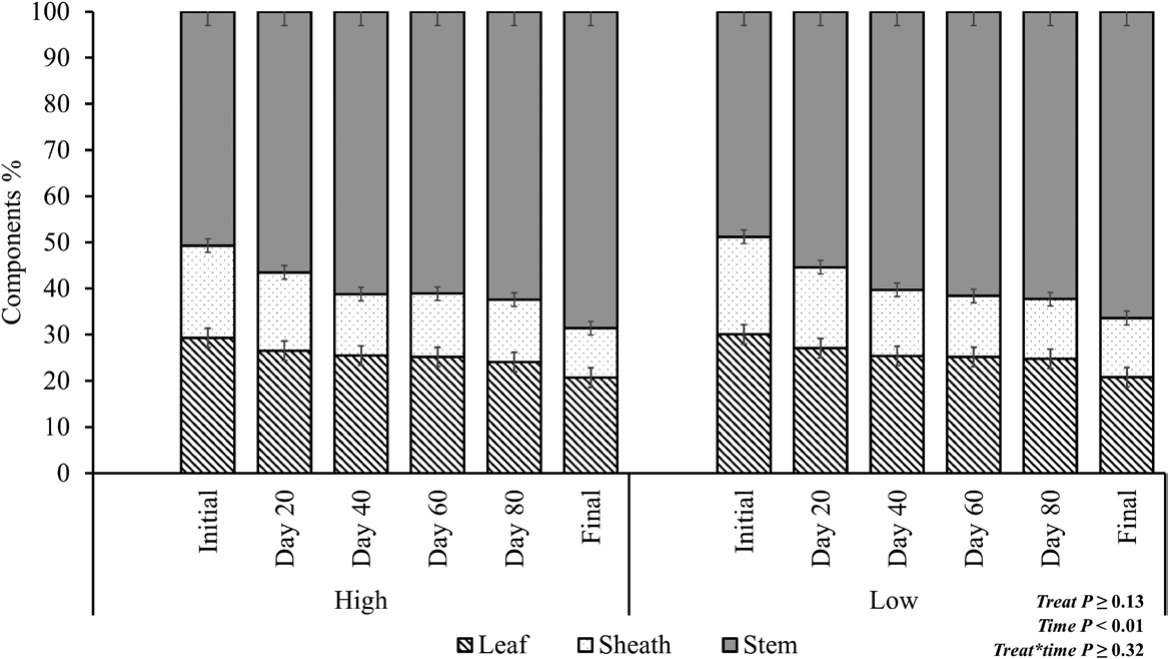
Effects of stocking rate and period (0, 20, 40, 60, 80, and 96 d) on residue components; LS, low stocking rate (3.5 AU/ha); HS, high stocking rate (7.0 AU/ha). Error bars represent the SEM.

There was no stocking rate × time interaction effect (*P* ≥ 0.32; Figure 4) on the change of proportion of morphology components in the mass of residue over time. There was also no SR effect (*P* ≥ 0.13; Figure 4). A time effect (*P* < 0.01) was observed with % leaves and % sheath decreasing over time (*P* < 0.01), with an average of 29.3% and 22.3%, respectively at the beginning of the experimental period and 19.6% and 11.5% at the end of the experimental grazing period, respectively. Conversely, the % stem increased over time, with an average of 46.8% at the initial, and 71.3% at the end of the experimental period.

### Animal behavior

**Figure 5.**
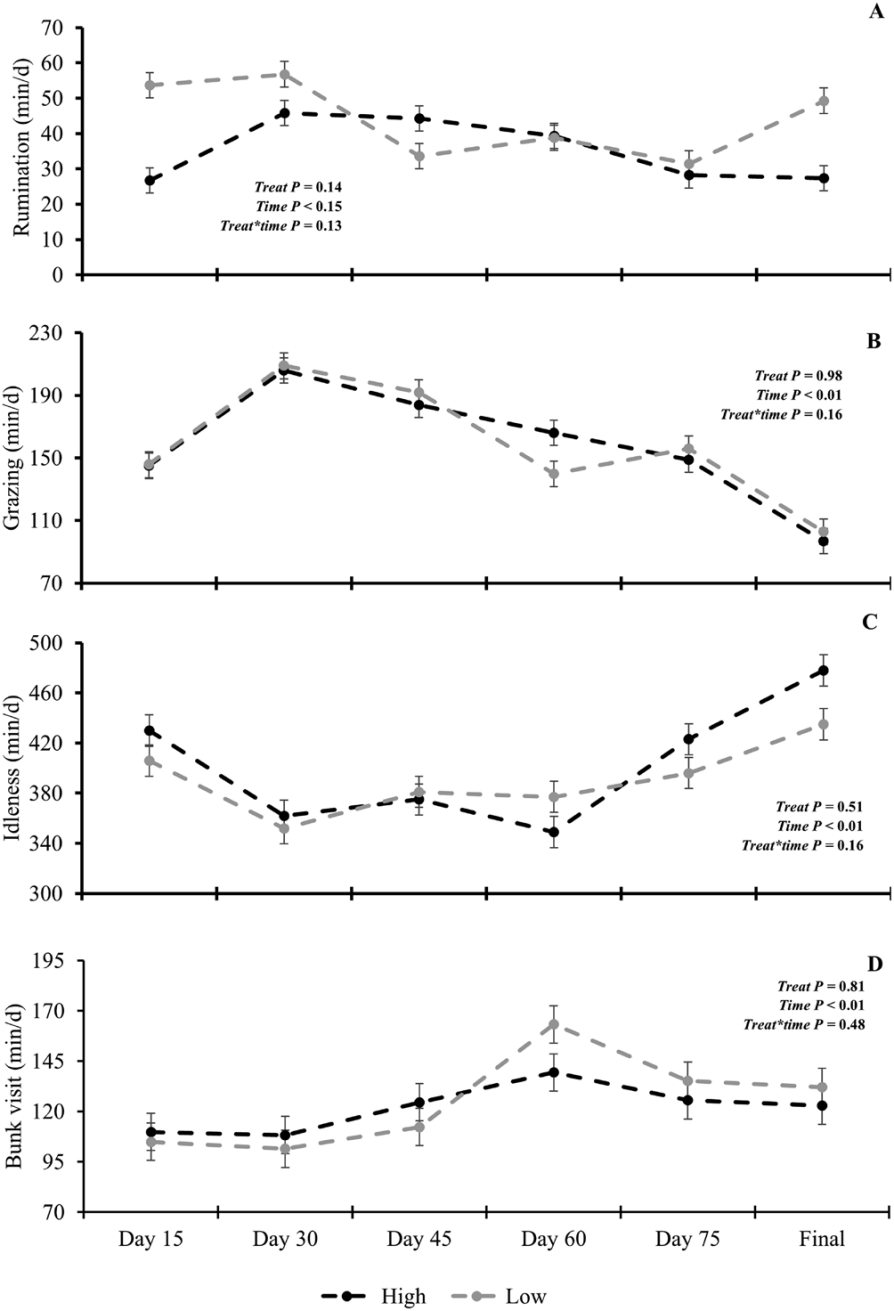
Effects of stocking rate and period (0, 20, 40, 60, 80, and 96 d) on animal behavior; A = rumination; B = grazing; C = Idleness; D = bunk visit; LS, low stocking rate (3.5 AU/ha); HS, high stocking rate (7.0 AU/ha). Error bars represent the SEM.

There was no SR × time effect (*P* ≥ 0.16; Figure 5) for behavior characteristics, and there were no differences (*P* ≥ 0.15) in feed bunk visits, grazing, rumination, and idleness between SR. There was also no time effect (*P* = 0.16; Figure 5A) for time spent in rumination.

Grazing (*P* < 0.01, Figure 5B), increased from days 15 to 30 (145 vs 206 min/d) and then decreased after that until the end of the experimental period (day 96) when 97 min/d was observed. Conversely, time spent in idleness and feed bunk visits increased over time (*P* < 0.01; Figure 5C), although idleness decreased on days 30, 45, and 60 compared to day 15 (418 vs 357 min/d) after this period the time in this activity increased to 457 min/d at the end of the experimental grazing period (day 96). Feed bunk visits (Figure 5D) were similar until day 45 of the experimental period but increased on day 60 until the end of the experimental period.

### Nutritive Value

There were no treatment × time interactions detected (*P* ≥ 0.68; Table 3) for the nutrient composition of residue over time. There were also no differences (*P* ≥ 0.19) in OM, apNDF, CP, or iNDF between treatments. However, a time effect for OM, apNDF, CP, or iNDF was detected (*P* < 0.01). The OM, apNDF, and iNDF increased over time, with the initial period (92.4%, 75.8%, and 42.1%, respectively) having lesser OM, apNDF, and iNDF than the final period (93.6%, 77.6%, and 43.1%, respectively). Conversely, the CP decreased over time, with 4.1% in the initial period compared to 3.6% at the end of the experimental period.

**Table 3.** Effect of stocking rate of finishing heifers grazing snaplage residue and fed ad libitum concentrate on nutrient composition of the residue over time

Item	Treatments^†^		SEM^*^	*P-value*		
	HS	LS		treat	time	treat × time
Organic matter, %						
Initial	92.1	92.8	0.44	0.19	<0.01	0.97
Day 20	92.6	93.4				
Day 40	92.7	93.5				
Day 60	92.7	93.6				
Day 80	92.8	93.6				
Final	93.3	93.9				
apNDF^‡^, %						
Initial	74.9	76.7	1.02	0.31	<0.01	0.75
Day 20	76.1	77.1				
Day 40	76.2	77.7				
Day 60	76.3	77.8				
Day 80	76.3	78.0				
Final	77.0	78.3				
iNDF^‖^, %						
Initial	43.3	40.9	1.06	0.40	<0.01	0.68
Day 20	42.5	41.6				
Day 40	43.1	41.8				
Day 60	43.2	41.8				
Day 80	43.2	41.8				
Final	43.8	42.3				
Crude protein, %						
Initial	4.0	4.2	0.13	0.32	<0.01	0.97
Day 20	3.8	4.0				
Day 40	3.8	3.9				
Day 60	3.7	3.9				
Day 80	3.7	3.8				
Final	3.6	3.7				

^*^Standard error of the means.

^†^Treatments: HS (High stocking rate; 7.0 AU/ha), LS (Low stocking rate; 3.5 AU/ha).

^‡^apNDF: Neutral detergent fiber corrected for ash and protein.

^‖^iNDF: Indigestible Neutral detergent fiber (incubated in the rumen for 288 h).

### Intake and Apparent Digestibility

There were no differences (*P* ≥ 0.65; Table 4) in the apparent digestibility of DM, OM, CP, NDF, starch, or EE between treatments. There were no treatment effects (*P* ≥ 0.40; Table 5) for total intake, concentrate intake, and residue intake with LS (2.89%, 2.72%, and 0.172% of BW/d) and HS (2.84, 2.69, and 0.151% of BW/d). There were no differences (*P* ≥ 0.14; Table 5) in the intake of OM, CP, NDF, starch, or EE between treatments. No differences between SR were observed for forage DOMI and CP/DOM ratio (*P* = 0.44 and *P* = 0.88, respectively).

**Table 4.** Effect of stocking rate of finishing heifers grazing snaplage residue and fed ad libitum concentrate on apparent digestibility

Item	Treatments^†^		SEM^*^	*P-value*
	HS	LS		
DM, %	80.8	79.2	2.37	0.39
OM, % DM	80.8	79.7	2.31	0.44
CP, % DM	82.8	82.0	1.67	0.61
apNDF, % DM	35.0	35.8	4.29	0.89
Starch, % DM	92.2	91.5	1.57	0.43
EE, % DM	62.2	60.1	3.87	0.68

^*^Standard error of the means.

^†^Treatments: HS (High stocking rate; 7.0 AU/ha), LS (Low stocking rate; 3.5 AU/ha).

DM, dry matter; OM, organic matter; CP, crude protein; apNDF, neutral detergent fiber corrected to ash and protein; EE, ether extract.

**Table 5.** Effect of stocking rate of finishing heifers grazing snaplage residue and fed ad libitum concentrate on intake as a percent of BW

Item	Treatments^†^		SEM^*^	*P-value*
	HS	LS		
Total, % BW/d	2.84	2.89	0.07	0.61
Residue, % BW/d	0.151	0.172	0.027	0.40
Concentrate, % BW/d	2.69	2.72	0.091	0.77
OM, % BW/d	2.39	2.53	0.061	0.16
CP, % BW/d	0.421	0.445	0.012	0.19
apNDF, % BW/d	0.20	0.21	0.022	0.36
Starch, % BW/d	1.90	1.99	0.043	0.14
EE, % BW/d	0.098	0.101	0.001	0.42
DOMI, kg/d	5.50	5.77	0.243	0.44
CP/DOM, g/kg	222	223	5.3	0.88

^*^Standard error of the means.

^†^Treatments: HS (High stocking rate; 7.0 AU/ha), LS (Low stocking rate; 3.5 AU/ha).

OM, organic matter; CP, crude protein; apNDF, neutral detergent fiber corrected to ash and protein; EE, ether extract; DOMI, digestible organic matter intake; CP/DOM, crude protein/digestible organic matter ratio.

### Performance

No differences between SR were observed (*P *≥ 0.88; Table 6) in average daily gain, initial or final BW. Furthermore, there was no difference (*P *≥ 0.34) between treatments for the residue, concentrate, or total dry matter intake. The HS had a 100% greater (*P *< 0.01) gain per area than the LS treatment (618 vs 309 kg/ha, respectively).

**Table 6.** Effect of stocking rate of finishing heifers grazing snaplage residue and fed ad libitum concentrate on heifer growth performance

Item	Treatments^*^		SEM	*P-value*
	HS	LS		
Body weight, kg				
Initial body weight	276.0	275.6	7.60	0.88
Final body weight	365.0	364.2	14.91	0.93
Average daily gain, kg/d	0.952	0.956	0.132	0.94
Dry matter intake, kg/d				
Residue,	0.434	0.497	0.060	0.34
Concentrate,	7.72	7.78	0.381	0.84
Total,	8.15	8.27	0.322	0.66
Feed efficiency, G:F	0.114	0.112	0.011	0.76
Gain per area, kg/ha	618	309	54	<0.01

^*^Treatments: HS (High stocking rate; 7.0 AU/ha), LS (Low stocking rate; 3.5 AU/ha).

SEM, standard error of means.

### Carcass Characteristics and Chemical Composition

No differences between SR were observed for hot carcass weight and dressing percentage (*P ≥ *0.35, Table 7). There were no differences in 12th rib fat thickness, Longissimus muscle area, and marbling score detected (*P* ≥ 0.12). No differences between SR were observed (*P ≥ *0.13) in initial or 24 h pH and temperature. The meat’s chemical composition was not different (*P *≥ 0.37) between treatments.

**Table 7.** Effect of stocking rate of finishing heifers grazing snaplage residue and fed ad libitum concentrate on carcass characteristics and chemical composition

Item	Treatments^*^		SEM	*P-value*
	HS	LS		
Hot carcass weight, kg	190.9	191.6	8.54	0.92
Dressing percentage. %	52.2	52.7	0.35	0.35
12th rib fat thickness, mm	3.12	3.59	0.312	0.12
Longissimus muscle area, cm²	63.3	62.0	1.23	0.39
Initial carcass pH	6.29	6.34	0.034	0.40
24h carcass pH	5.82	5.87	0.021	0.13
Initial Temperature (°C)	31.7	31.4	0.60	0.71
24h temperature (°C)	7.3	7.1	0.22	0.30
Marbling score^†^	366	373	5.8	0.43
Chemical composition				
Collagen, %	1.24	1.23	0.051	0.83
Protein, %	22.1	22.0	0.10	0.38
Fat, %	2.21	2.46	0.204	0.39
Moisture, %	71.3	71.1	0.17	0.37
Ash, %	3.11	3.58	0.095	0.42

^*^Treatments: HS (High stocking rate; 7.0 AU/ha), LS (Low stocking rate; 3.5 AU/ha).

^†^Calculated according to Dow et al. (2011).

SEM, standard error of means.

### Meat Quality

There were no SR × time interactions detected (*P* = 0.30; Table 8) for cooking loss, L*, a*, b*, C*, and, h*. There were also no differences (*P* ≥ 0.32) among SR for these variables. However, a time effect was detected (*P* < 0.01). For cooking loss, there was a 13.6% reduction on day 14 compared to day 0. On the other hand, the maturation process increased over time (*P* < 0.04) L*, b*, C*, and, h*.

**Table 8.** Effect stocking rate of finishing heifers grazing snaplage residue and fed ad libitum concentrate on cooking losses and color

Item	Day 0		Day14		SEM^a^	*P-value*		
	HS	LS	HS	LS		treat^†^	time	treat × time
Cooking losses, g	22.1	22.6	18.9	19.7	0.72	0.41	<0.01	0.78
L*	43.0	43.5	44.2	44.5	0.42	0.35	<0.01	0.78
a*	20.4	20.1	19.7	19.7	0.61	0.76	0.256	0.82
b*	13.3	13.2	15.4	14.8	0.29	0.33	<0.01	0.30
C*	24.4	23.9	24.9	25.3	0.55	0.98	0.05	0.34
h*	33.8	33.4	38.6	37.4	0.73	0.56	<0.01	0.36

^†^Treatments: HS (High stocking rate; 7.0 AU/ha), LS (Low stocking rate; 3.5 AU/ha).

SEM, standard error of means.

## Discussion

The capacity of ruminants to use byproducts, crop residues, and feeds inappropriate for human nutrition has been increasingly explored within the production systems (Russelle et al., 2007; Redfearn et al., 2019). Still, crop residues have their particularities, especially undergrazing. Animal productivity in a grazing system is directly linked to the variables of animal performance and stocking rate (Jones and Sandland, 1974; Pereira et al., 2020). The traditional GFS maximizes individual performance, once the concentrate becomes the main feed source. It provides the protein, energy, and mineral supply required for the finishing phase, with grass as a source of fiber for maintaining rumen health. However, the stocking rate can vary because the high amount of supplementation offered promotes a substitution effect (Moore et al., 1999; Paterson et al., 2015). This effect allows for an increase in the stocking rate.

The grazing system has constant sprouting, growth, and senescence. This process results in the herbage mass, which is a balance between forage input (i.e., forage growth) and forage output (i.e., forage senescence and offtake; Homem et al., 2021). By understanding these dynamics, it is possible to make correct adjustments to the stocking rate. However, when under SRFS, there is a considerable reduction in the residue availability over time, as indicated by the results of residue mass. This reduction occurs due to grazing, trampling, and weather factors (Russell et al., 1993; Stalker et al., 2015; Lehman et al., 2021). Lehman et al. (2021) observed a reduction of 22% of the initial mass of corn plant residue by weathering from September to November. Also, they found a loss by grazing (~ 50%). Other studies (Stalker et al., 2015; Ulmer et al., 2019), reported similar losses due to weathering but at different times of the year (November to February). However, these authors found a reduction in residue mass of approximately 25% by grazing. These differences in grazing disappearance can be explained by differences in stocking rates, length of the grazing period, and weather during grazing (Ulmer et al., 2019). In the current experiment, a 46% reduction was observed for HS with an initial mass of 9,659 kg/ha pre-grazing and 5,152 kg/ha post-grazing. The availability of residue in LS was 9,685 kg/ha pre-grazing and 6,434 kg/ha post-grazing, resulting in a reduction of 33%.

The residue availability is important to be determined since the amount of residue from snaplage in the field can vary due to the hybrid chosen, weather conditions inherent to the production system and location, season or time of year, and different harvesting equipment (Ferraretto et al., 2018; Lima et al., 2022). Although the availability of residue in the LS at the end of the experiment is similar to the HS at the beginning of the experiment, the quality of the residue is different since the final residue is composed of less digestible material due to the grazing pressure, animal grazing, and weather factors.

The traditional corn plant residue is characterized by leaf, sheath, husk, stem, and eventual remaining cobs, while the residue of snaplage is characterized by leaf, sheath, and stem. In both residues, the leaf is the component with a faster disappearance either to climatic factors affecting degradation or grazing (Stalker et al., 2015). In their previous research, Stalker et al. (2015) observed that at a stocking rate of 5.0 AU/ha, husks and leaves were reduced by 82% and 47%, respectively. Whereas, at 2.5 AU/ha treatment, these components were reduced by 57% and 42%, respectively, after grazing. The stem mass remained the same after grazing, regardless of the stocking rate used, demonstrating that cattle avoid consuming stems. However, in snaplage residue, where the availability of more digestible material is lower, animals are forced to consume a less digestible portion of the plant (stem).

The nutrient contents did not change over time in components of the plant when analyzed and compared individually as observed by Stalker et al. (2015). They found similar values of ash, NDF, and IVOMD before and after grazing for all components. Furthermore, similar CP content was found at both sampling dates for all plant parts, except the husk. Fernandez-Rivera and Klopfenstein (1989), evaluated grazing under differing stocking rates (2.47 and 4.69 AU/ha), and found that the CP content of corn residue was not different before (4.7% and 4.9% CP) and after grazing (5.1% and 4.7% CP) for the stocking rates evaluated, respectively. In addition, no differences were observed in the CP content of leaf and husk, 5.6% before and 4.8% after grazing. However, the disappearance of more digestible components of the corn residue either by weathering or grazing selectivity results in a change in the nutritive value of the diet (Lamm and Ward, 1981; Fernandez-Rivera and Klopfenstein, 1989; Stalker et al., 2015; Lehman et al., 2021). Lamm and Ward (1981) investigated changes in the composition of corn crop residues and observed a decline in CP (8.8% vs 8.2%) and IVOMD (72.0% and 59.2%) for pre-grazing and post-grazing, respectively. Similarly, Lehman et al. (2021) found no differences in NDF, CP, or OM among treatments pre-grazing. However, in post-grazing data, grazed paddocks had increased ADF and decreased CP compared with ungrazed paddocks. Although Lehman et al. (2021) did not perform statistical analysis, it is possible to note an increase in average NDF (74.4% vs 78.2%) and ADF (74.4% vs 78.2%) and a decrease in CP (3.9% vs 3.4%) and OM (92.3% vs 87.0%) when comparing pre-grazing and post-grazing, respectively, in all treatments evaluated. This could explain the change in the diet over time in the current experiment. The increase of stem proportion in the residue mass increased OM, apNDF, iNDF content, and a decrease in CP content.

The greater residue disappearance in HS did not lead to changes in behavioral data compared to LS. However, the time spent grazing decreased, and idleness and feed bunk visits increased over time. These results could be explained by the change in the proportion of morphological components of the residue. Animal grazing selectivity and weathering were likely the main factors for the difference in the proportion of components over time. Although the increase in feed bunk visit time could be an explanation for the reduction in grazing time, only an increase of 20 min spent on this activity was observed, whereas the decrease in grazing time was 90 min. Therefore, the decrease in time spent on grazing activity after day 30 until the end of the experimental period could be explained by changes in the residue characteristic. The different nutritional values and digestibility of residue components likely led the animals to initially seek greater digestibility components (Fernandez-Rivera and Klopfenstein, 1989; Gutierrez-Ornelas and Klopfenstein, 1991; Stalker et al., 2015; Lehman et al., 2021). Thus, with the disappearance of the more digestible components, the animal likely does not spend as much time looking for more digestible material as the area is more uniform, predominantly composed of stem or sheath attached to the stem. In addition, less time grazing can be explained by the roughage in this system acting only to maintain rumen health. The stem component is characterized by low-quality material and high content of NDF, acting as a source of physically effective fiber (peNDF) stimulating rumination and saliva production (Mertens, 1997). Thus, the animal needs less material to reach the level needed to maintain rumen function resulting in no difference in rumination time (Figure 5d) observed with reduced grazing time and an increase in idleness time (Figure 5c).

Increasing the stocking rate from 3.5 to 7 AU/ha was not enough to alter the performance. The same amount of concentrate was available for both treatments. In the GFS, animal performance depends on the sex of the animal, level of supplementation, and season of the year (dry and rainy seasons). In the current study, heifers were used in a system where forage quantity and quality decreased over time, and the animals were not adapted to the supplementation. Hence, the ADG can be considered reasonable (0.96 kg/d). The fact that concentrate was fed ad libitum, could lead to acidosis as they consume diets rich in rapidly fermenting carbohydrates (Owens et al., 1998). In addition, no adaptation diet was used as the aim was to challenge the animals to the potential of limited roughage availability. When adaptation is eliminated or the diet changes rapidly, the animals are predisposed to the risk of acidosis (Goad et al., 1998; Coe et al., 1999). It was therefore expected that a reduction in the amount of snaplage residue available in HS treatment would result in increased acidosis, thereby affecting performance. However, both treatments had a similar proportion of components in the mass of the residue. Furthermore, we hypothesized that the leaf would be selected first in the HS treatment, leading to a reduction in performance over time when compared to the LS treatment because the leaf is more of a nutritive and digestible material than the stem and sheath. Thus, an increasing stocking rate would result in a more rapidly increasing intake of this material by animals in the HS treatment. However, the lower mass in the HS treatment did not result in lower leaf proportion since the disappearance of most digestive morphological components happened proportionally in both treatments.

The similarity of the diet in both treatments led to similar digestibility, nutrient intake, and residue intake per body weight percentage since the residue was not limiting in both treatments, with 5,162 and 6,434 kg DM/ha remaining in the HS and LS treatments, respectively, at the end of the experimental period. Thus, the animals performed similarly in both stocking rates because, although consuming low-quality material, the residue represented approximately 5% of the diet with an intake of less than 0.2% of BW, resulting in similar feed efficiency. Our results indicate that increasing the stocking rate from 3.5 AU/ha to 7 AU/ha without affecting individual animal performance increases gain per area. The HS had the greatest bodyweight gain per area, with an increase of 100% compared with the LS. Increasing the stocking rate allows you to double the number of animals and maximize the use of land and residue without affecting the quality of the finished carcass since there was no change in the chemical and physical characteristics data.

We hypothesized differences in meat quality due to a possible acidosis in the HS treatment since acidosis can affect marbling and fatty acid profile in meat from ruminants (Ladeira et al., 2018). The similarity in performance was also reflected in the meat quality results. However, the higher stocking rate did not limit residue or decrease animal performance. Thus, it is unsurprising that nearly all meat quality parameters were not affected by the stocking rate.

## Conclusion

The data presented in this study demonstrate that there is an opportunity for expanding snaplage residue use through grazing, especially on farms where the residue is not used as a cover crop and beef cattle is the main activity. Beef cattle producers can adopt stocking rates ranging from 3.5 to 7.0 UA/ha. However, a greater stocking rate (7.0 UA/ha) improves gain per area, without affecting performance and carcass traits.
